# Application of next-generation sequencing to characterize novel mutations in clarithromycin-susceptible *Helicobacter pylori* strains with A2143G of 23S rRNA gene

**DOI:** 10.1186/s12941-018-0259-8

**Published:** 2018-03-22

**Authors:** Jiaoe Chen, Liping Ye, Liangmin Jin, Xuehua Xu, Peisong Xu, Xianjun Wang, Hongzhang Li

**Affiliations:** 1 Department of Gastroenterology, Sanmen People’s Hospital, No. 117, Renmin Road, Sanmen, 317100 Zhejiang People’s Republic of China; 2 Department of Gastroenterology, Zhejiang Taizhou Hospital, Taizhou, 31700 People’s Republic of China; 3 Department of Research Service, Zhiyuan Inspection Medical Institute, Hangzhou, Zhejiang 310006 People’s Republic of China; 4 Clinical Laboratory, Hangzhou First People’s Hospital, Hangzhou, 310006 Zhejiang People’s Republic of China

**Keywords:** *Helicobacter pylori*, Clarithromycin, Susceptible strains, 23S rRNA, SNVs, RND family

## Abstract

**Background:**

Clarithromycin (CLR) resistance has become a predominant factor for treatment failure of *Helicobacter pylori* eradication. Although the molecular mechanism of CLR resistance has been clearly understood in *H. pylori*, it is lack of evidence of other genes involved in drug resistance. Furthermore, the molecular mechanism of phenotype susceptible to CLR while genotype of 23S rRNA is mutant with A2143G is unclear. Here, we characterized the mutations of CLR-resistant and -susceptible *H. pylori* strains to explore bacterial resistance.

**Methods:**

In the present study, the whole genomes of twelve clinical isolated *H. pylori* strains were sequenced, including two CLR-susceptible strains with mutation of A2143G. Single nucleotide variants (SNVs) were extracted and analyzed from multidrug efflux transporter genes.

**Results:**

We did not find mutations associated with known CLR-resistant sites except for controversial T2182C outside of A2143G in the 23S rRNA gene. Although total SNVs of multidrug efflux transporter gene and the SNVs of HP0605 were significant differences (P ≤ 0.05) between phenotype resistant and susceptible strains. There is no significant difference in SNVs of RND or MFS (HP1181) family. However, the number of mutations in the RND family was significantly higher in the mutant strain (A2143G) than in the wild type. In addition, three special variations from two membrane proteins of *mtrC* and *hefD* were identified in both CLR-susceptible strains with A2143G.

**Conclusions:**

Next-generation sequencing is a practical strategy for analyzing genomic variation associated with antibiotic resistance in *H. pylori*. The variations of membrane proteins of the RND family may be able to participate in the regulation of clinical isolated *H. pylori* susceptibility profiles.

## Background

*Helicobacter pylori* (*H. pylori*), a Gram-negative and microaerophilic bacterium, has been recognized an important human pathogen that infects approximately 50% of world’s population, and is responsible for the development of upper gastrointestinal disorders, including chronic gastritis, peptic ulcer disease, gastric cancer and mucosa-associated lymphoid tissue (MALT) lymphoma [1–3]. In the past few decades, triple therapy regimen consist of a proton pump inhibitor in combination with two antibiotics, such as clarithromycin (CLR) and amoxicillin (AMX) or metronidazole (MTZ), which has been recommended as first-line treatment regimen for *H. pylori* infection [4]. However, with the increase of *H. pylori* CLR-resistant strains, this traditional treatment regimen is being replaced by quadruple therapy or precise medical, especially in the area of CLR resistance is higher than 15% [5–7]. Many reports have indicated that CLR resistance has become a predominant factor for treatment failure in which containing CLR [5, 8].

The majority of *H. pylori* CLR-resistant strains present three point mutations in the region of domain V of 23S ribosomal RNA (rRNA): A2142G, A2142C and A2143G. Simultaneously, the studies also suggest that some other point mutations may be involved in CLR resistance at position 2115G, G2141A, T2117C, T2182C, T2717C [9–11]. Another mechanism of resistance to CLR has been reported that five conserved families of multidrug efflux pump transporter contribute to bacterial antibiotic resistance. One of these, the resistance-nodulation-cell division (RND) family, was consisted of an inner membrane efflux protein, a membrane fusion protein and an outer membrane protein. Currently, four gene clusters (HP0605–HP0607, HP0969–HP0971, HP1327–HP1329, HP1487–HP1489) [12–15] have been established as RND family candidates in *H. pylori*. Hirata et al. reported that the MIC of CLR-resistant strains was decreased by using efflux pump inhibitor (EPI), indicating that in addition to the point mutation of 23S rRNA gene, the efflux pump cluster is also involved in the development of resistance to CLR [16].

Although the molecular mechanism of CLR resistance has been relatively clearly understood in *H. pylori*, it is unclear whether other gene mutations associated with CLR resistance outside 23S rRNA and RND family. With increase of the CLR-resistant strains, several studies have suggested that other genetic factors could be involved in the increased antibiotic resistance. Recently, Binh et al. [17] revealed that mutations of insertion or deletion in *rpl*22 and guanine to adenine point mutation at position 60 in *inf*B gene could be related to CLR resistance using whole-genome sequencing of induced CLR-resistant strains in vitro.

However, almost all of the researches were focus on exploring potential antibiotic resistance genes. Until now, to the best of our knowledge, there is very little research on antibiotic susceptibility gene. In our previous research, we isolated two strains that were identified adenine to guanine mutations at position 2143 in 23S rRNA, while the outcome of antibiotic susceptibility testing were susceptibility to CLR or lower CLR resistance, suggesting that there were some other genes participated in the regulation of antibiotic resistance or susceptibility to CLR.

Compared with traditional DNA sequencing, next-generation sequencing (NGS) is a revolution of sequencing technology that can be sequencing millions of DNA molecules massively parallel in less time and at lower cost [18, 19]. To date, more than three thousand microbial genome sequences have been completed and published by using NGS according to the report [20, 21]. Now, NGS has also been used to identify bacterial single nucleotide polymorphisms (SNP) or mutation associated with antibiotic resistance [22–24]. Although we didn’t identify all of sequencing information that is like a needle in a haystack because a large number of redundant data is generated by the NGS, with the development of sequencing and biological information, the genetic code will be eventually cracked one by one.

In this study, to characterize the multidrug efflux transporter gene variants in the CLR genotype-resistant while phenotype-susceptible strains, we applied Sanger sequencing to detect the genotype of 23S rRNA and NGS to analysis of genomic variation in clinical isolated *H. pylori* strains. CLR susceptibility testing was performed by E-test and agar dilution. Ten HP strains meeting our requirements were applied to whole-genome sequencing, including four CLR-resistant and six CLR-susceptible strains.

## Methods

### Isolation and culture of *H. pylori*

Gastric mucosa tissue samples were collected from patients with upper gastrointestinal disease during endoscopy at Sanmen People’s Hospital and Zhejiang Taizhou Hospital. Isolation and culture of *H. pylori* were performed at the laboratory of Hangzhou Zhiyuan Medical Inspection Institute. Patients were investigated to have not taken any antibiotics for at least 4 weeks before examination. This study had received a strict medical ethics review, and written informed consent was obtained from every patient.

The isolation and identification of *H. pylori* were performed as described in previous studies [25, 26]. Gastric mucosa homogenate tissue was transferred onto a Columbia agar plates containing 5% fresh defibrinated sheep blood and cultured under microaerophilic conditions (5% O_2_, 10% CO_2_ and 85% N_2_) at 37 °C for 3–7 days. Suspicious colonies were confirmed by Gram stain, urease, oxidase, and catalase activity testing.

### Antibiotic susceptibility testing

The antibiotic resistance of *H. pylori* to CLR was performed by E-test and agar dilution methods according to the protocols of the Clinical and Laboratory Standards Institute (Wayne, PA, USA) [27]. Briefly, the concentration of *H. pylori* was regulated with saline to a 2.0 McFarland standard, and the suspensions were inoculated onto Mueller–Hinton agar plate supplemented with 5% sheep blood. The CLR E test strip was attached on the plate and incubated at 37 °C for 3–5 days under microaerophilic conditions. Agar dilution was performed by serial twofold dilutions of CLR. The breakpoint of CLR resistance was ≥ 1 mg/l. ATCC43504 (NCTC11637) was used as the control strain and all tests were performed by Hangzhou Zhiyuan Medical Inspection Institute.

### Direct sequencing characterized the mutations of 23S rRNA

According to the reference sequence of HP U27270, HP-23S forward primer (5′-ATGAATGGCGTAACGAGATG-3′) and HP-23S reverse primer (5′-ACACTCAACTTGCGATTTCC-3′) were employed to detect 23S rRNA gene mutations at positions of 2142 and 2143. The PCR reaction was performed in 25-µl reactions containing 2.5 µl of 10× LA Taq Buffer, 4 µl of dNTP mixture (2.5 mM each), 0.5 µl each 10 µM primer, 2 µl template DNA and 0.25 µl of TaKaRa LA Taq™ (5 units/µl). The parameters of PCR were carried out at 94 °C for 5 min, followed by 25 cycles of denaturing at 94 °C for 30 s, annealing at 58 °C for 30 s, and extending at 72 °C for 3 min, with a final extension for 10 min at 72 °C. 1.2% agarose gel electrophoresis was utilized for verifying the PCR products size at 360 bp. To validate the mutations of 23S rRNA, Sanger sequencing was performed with an ABI 3730XL DNA Analyzer (Applied Biosystems, Foster City, CA, USA) using BigDye^®^ Terminator V3.1 according to the manufacturer’s instructions.

### DNA extraction and whole-genome sequencing

The total genomic DNAs of *H. pylori* were extracted by using Invitrogen Purelink Genomic DNA Mini Kit (Life Technologies, Carlsbad, CA, USA) according to the manufacturer’s instructions. The concentration of each genomic DNA sample was quantified with Qubit dsDNA HS assay kit (Life Technologies). For each sample, 1 μg genomic DNA was randomly uniformly fragmented to < 500 bp by sonication (Diagenode Bioruptor UCD-200) and the library was prepared by using NEB Next^®^ Ultra™ DNA Library Prep Kit for Illumina^®^ following the manufacturer’s protocol. Quality control of the library was identified by Agilent 2100 Bioanalyzer (Agilent Technologies, Palo Alto, CA, USA) with DNA 1000 chip according to the manufacturer’s instructions. Whole-genome sequencing was performed with the Illumina HiSeq (Illumina, San Diego, CA, USA) platform to generate 2 × 150-bp paired-end reads. Image analysis and base calling were conducted by the HiSeq Control Software (HCS) and GAPipeline-1.6 (Illumina) on the HiSeq instrument.

### Genome analysis and single nucleotide variations (SNVs) calling

To reduce the false discovery rate of SNVs, the low-quality bases (the quality of both ends bases < Q20) and raw reads (> 10 N bases and reads lengths < 75 bp) were trimmed by Trimmomatic (version 0.30). The remaining clean reads were mapped against the reference genome with GenBank accession NC_000915 and CP003904 using BWA [28] (version 0.7.12). SNVs and InDel were called using SAMTOOLS’s [29] (version 1.1) Mpileup module and Bcftools with default parameters, and finally a series of mutations were generation.

### Variations of multidrug resistance genes

In order to validate the role of multidrug efflux transporter gene in CLR-resistant and CLR-susceptible *H. pylori*, the variations of RND family, major facilitator superfamily (MFS) and adenosine triphosphate (ATP)-binding cassette (ABC) superfamilies had been analyzed, including HP0605–HP0607, HP0969–HP0971, HP1327–HP1329, HP1487–HP1489, HP1181, HP1184, HP0600, HP0613, HP0759, HP1082, HP1206, HP1220, HP1321 and HP1486.

### Statistical analysis

All statistical analyses were conducted with SPSS statistical software package version 19.0 (SPSS Inc., Chicago, IL, USA). The relationships between variations of multidrug resistance genes and CLR resistance/susceptibility were investigated by Student’s t-test. A P value ≤ 0.05 was considered statistically significant.

## Results

### Genotype of 23S rRNA and phenotype of *H. pylori* resistant to CLR

**Fig. 1:**
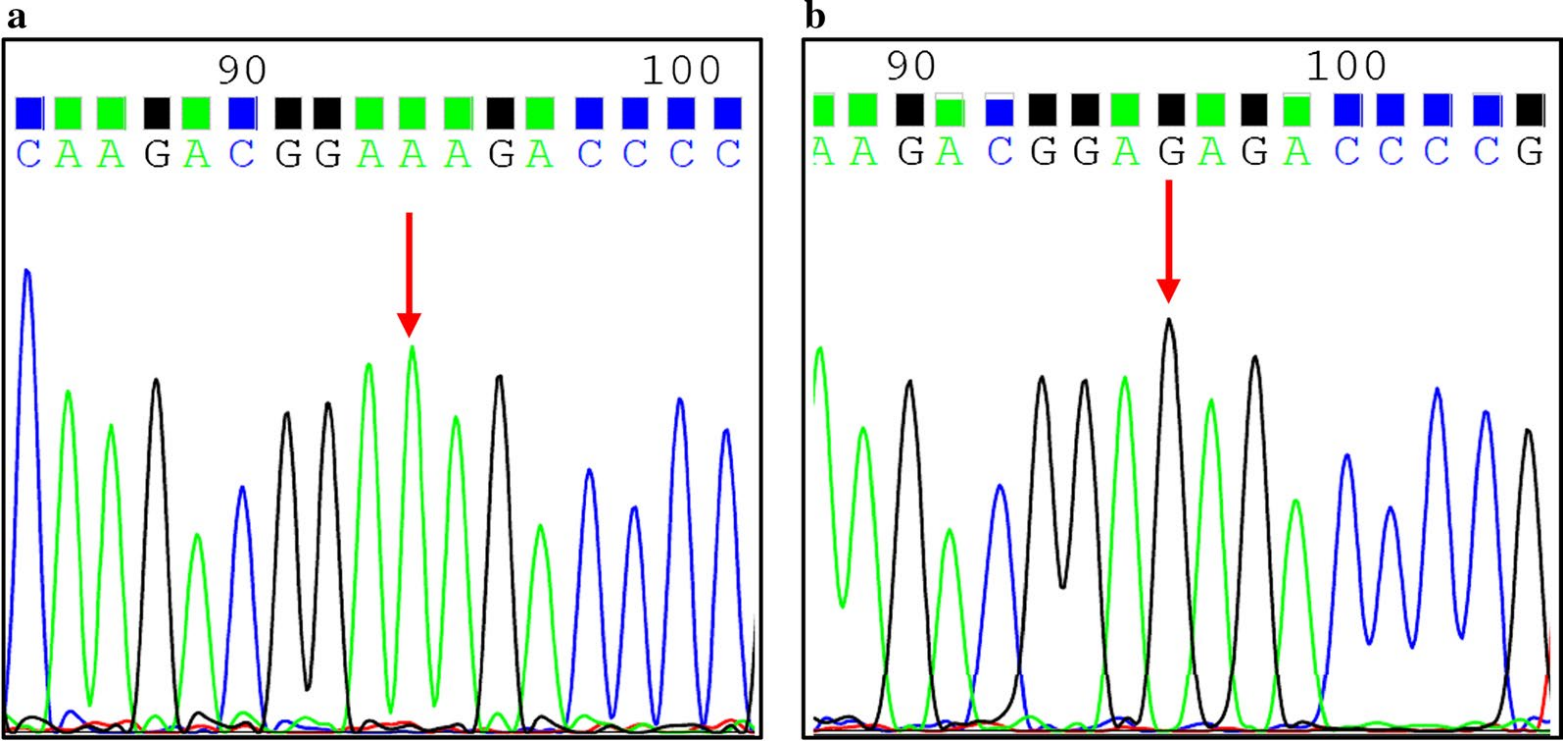
The results of Sanger sequencing for genotype of 23S rRNA in *H. pylori*. **a** The wild type without mutation at position 2143. **b** The mutant strain with mutation of A>G at position 2143

For twelve clinical isolated strains, CLR susceptibility testing indicated that the six of strains were susceptible to CLR and six of strains were resistant to CLR. The genotype of 23S rRNA from *H. pylori* was verified by Sanger sequencing (Fig. 1). Eight of them were mutant with A2143G and four strains were wild type (Table 1). All of phenotype-resistant strains presented mutation A to G at position 2143 of the 23S rRNA. However, two mutant-type (S2, S3) of 23S rRNA gene at position 2143 (A>G) were also detected in six phenotype-susceptible strains.

**Table 1:** The results of whole-genome sequencing for each sample

Strain ID	CLR susceptibility testing	Total reads (clean reads)	Mapping to genome reads	Covered length	Coverage (%)	Average depth	Total SNVs	Total InDels
S1	R^a^	9,815,508	8,780,946	1,517,430	90.98	545.66	67,219	106
*S2*	*S* ^b^	*10,573,646*	*9,813,458*	*1,534,369*	*91.99*	*601.38*	*68,671*	*95*
*S3*	*S*	*10,269,810*	*9,529,590*	*1,527,013*	*91.55*	*565.38*	*70,135*	*101*
S4	R	9,931,112	8,921,164	1,553,847	93.16	522.64	67,929	132
S5	R	9,965,800	8,012,304	1,522,585	91.29	459.11	67,580	134
S6	S	8,326,490	7,203,062	1,537,013	92.15	528.19	68,032	99
S7	S	8,023,030	7,541,380	1,536,514	92.12	549.71	68,496	112
S8	R	7,687,812	7,171,310	1,522,399	91.28	535.58	69,891	104
S9	S	11,190,968	9805694	1,522,469	91.28	731.93	68,658	67
S10	S	12,167,520	11,643,382	1,539,435	92.3	888.02	70,933	94
S11	R	9,756,502	8,630,028	1,517,874	91.01	532.33	67,285	112
S12	R	6,575,708	6,090,808	1,521,421	91.22	378.89	67,231	177

S2 and S3 (italic values) indicates the phenotype-susceptible strains with mutant in A2143G of 23S rRNA gene

^a^R indicated the strain was resistant to clarithromycin

^b^S indicated the strain was susceptible to clarithromycin

### Overview of whole-genome sequencing of clinical isolated *H. pylori*

In the present study, twelve strains of *H. pylori* genome were successfully figured out by Hiseq sequencer. After trimming the low-quality reads, clean reads ranged from 6.6 to 12.2 million (Table 1). Clean reads were directly mapped to the reference genome. Coverage and average depth ranged from 90.98 to 93.16% and from 378 to 888, respectively. Therefore, the efficient reads were sufficient for subsequent analysis of SNVs. The number of SNVs and InDels ranged from 67,219 to 70,933 and from 67 to 177, respectively. There were no significant differences between CLR-resistant and CLR-susceptible strains in SNVs despite more prevalent in CLR-susceptible strains. However, the number of InDels was significantly decreased in CLR-susceptible strains.

### Identification of 23S rRNA gene mutations

To analyze the associations between phenotypic resistance and genotypic resistance, the mutations of the 23S rRNA gene were investigated. Consistent with Sanger sequencing, whole-genome sequencing indicated that six phenotype-resistant strains and two phenotype-susceptible strains had mutation A>G at position 2143. Simultaneously, the mutations outside of 2143 had been extracted (Table 2). Totally, we found fourteen mutations in addition to 2143. In this study, we did not find any mutations associated with known CLR-resistant sites except for T2182C and there is no significant difference between resistant strain and susceptible strain regardless of whether there was mutant with A2143G.

**Table 2:** SNVs of the 23S rRNA outside of 2143

Nucleotide position	Ref	Mutation	S1	*S2*	*S3*	S4	S5	S6	S7	S8	S9	S10	S11	S12
973	G	T	+	*−*	*−*	+	−	−	−	−	−	+	−	−
973	G	A	−	*−*	*−*	−	+	−	−	−	+	−	−	−
973	G	C	−	*+*	*+*	−	−	+	−	+	−	−	+	−
1023	G	A	+	*−*	*+*	−	−	−	−	−	+	−	−	−
1279	A	T	−	*−*	*−*	−	−	−	+	−	−	−	−	−
1280	A	G	−	*−*	*−*	−	−	−	+	−	−	−	−	−
1314	G	A	+	*−*	*−*	−	+	−	−	+	−	−	−	−
1513	G	A	+	*+*	*−*	−	−	−	+	−	+	−	−	−
2173	C	T	+	*−*	*−*	−	+	−	−	+	−	−	−	−
2182	T	C	+	*+*	*+*	+	+	+	−	+	+	+	+	+
2302	A	G	+	*−*	*−*	−	−	−	−	−	−	−	+	−
2485	T	C	−	*−*	*−*	−	+	−	−	−	−	−	−	+
2143	A	G	+	*+*	*+*	+	+	−	−	+	−	−	+	+

S2 and S3 (italic values) indicates the phenotype-susceptible strains with mutant in A2143G of 23S rRNA gene

+ Represented that the mutation had been detected and − represented the mutation had not been detected

### Identification of multidrug efflux transporter gene mutations

To characterize the mutations of multidrug efflux transporter genes, we focused on the study of the RND family, MFS and ABC superfamilies in *H. pylori*. Prior to identification of gene mutation in multidrug efflux genes, we removed the synonymous and InDels mutations in the CDS region. All mutations of these genes were presented in Table 3. Regardless of whether the strain is resistant to CLR, gene mutations were detected in all *H. pylori* of multidrug efflux transporter genes. The gene mutations of membrane fusion proteins (HP0606, HP0970, HP1328, HP1488) were significantly less than inner membrane proteins or outer membrane proteins in RND family.

**Table 3:** SNVs of multidrug efflux pump transporter genes

Gene	Strain											
	S1	*S2*	*S3*	S4	S5	S6	S7	S8	S9	S10	S11	S12
HP0605	21	*18*	*23*	24	23	16	20	27	15	17	29	27
HP0606	5	*5*	*6*	1	4	7	5	6	8	5	2	8
HP0607	38	*34*	*32*	32	33	30	22	31	32	32	27	36
HP0969	26	*23*	*25*	25	24	25	26	23	26	26	24	23
HP0970	7	*9*	*11*	7	7	9	8	8	8	9	9	7
HP0971	11	*15*	*12*	11	13	12	14	11	8	9	10	11
HP1327	22	*22*	*21*	24	22	22	21	22	23	25	23	22
HP1328	15	*12*	*15*	14	12	14	13	14	15	14	12	14
HP1329	28	*29*	*36*	30	28	25	33	30	26	24	32	30
HP1487	22	*21*	*15*	20	21	18	16	18	18	19	17	16
HP1488	7	*4*	*3*	6	4	4	5	6	5	4	4	5
HP1489	25	*28*	*23*	22	23	25	19	20	24	29	25	19
HP0600	50	*3*	*1*	52	48	23	20	46	5	31	47	44
HP0613	9	*7*	*6*	9	7	15	3	9	10	15	8	5
HP0759	10	*11*	*10*	10	11	11	8	10	11	9	8	11
HP1082	10	*13*	*12*	10	11	12	17	11	15	11	12	16
HP1181	10	*9*	*11*	9	10	7	11	12	12	9	9	12
HP1184	14	*19*	*14*	22	15	14	12	18	13	14	17	15
HP1206	13	*16*	*14*	13	14	13	27	16	16	18	18	12
HP1220	3	*3*	*4*	3	2	1	4	3	1	1	2	1
HP1321	26	*30*	*29*	22	24	29	32	30	24	24	30	28
HP1486	21	*18*	*18*	18	18	17	16	17	18	15	17	12
Total	393	*349*	*341*	384	374	349	352	388	333	360	382	374

S2 and S3 (italic values) indicates the phenotype-susceptible strains with mutant in A2143G of 23S rRNA gene

We didn’t find significant differences in gene mutations of RND or MFS (HP1181) families between CLR phenotype resistant strains and CLR phenotype susceptible strains. However, the total SNVs of multidrug efflux genes were significant differences between them. Unexpectedly, when we were grouped according to the 23S rRNA genotype, the number of mutations in the RND family was significantly higher in the mutant strain (A2143G) than in the wild type, and the difference was statistically significant. These results suggested the mutations from multidrug efflux genes may play an important role in CLR-resistance, and the mutations of the RND family may change the resistance of *H. pylori* to CLR with A2143G.

### Special variations in CLR-susceptible strains with A2143G

In the present study, we found two *H. pylori* strains were susceptible to CLR, while the genotypes of 23S rRNA were mutant with A2143G. To understand the cause of this discrepancy, analysis of the special variations in both strains was performed. The special variations were extracted by removing the mutations present in other CLR resistant strains with mutation of A2143G. Totally, 320 non-synonymous variations were obtained, and we selected 16 variations in 14 genes as putative susceptible gene (Table 4).

**Table 4:** The special variations were identified in CLA-resistant strain with A2143G

Gene	Position of mutation^a^	Ref	Mutation	Amino acid	Annotation	Mean depth
HP0254	265	G	A	G89S	Outer membrane protein HopG	465.5
rpsA	482	G	A	G161D	30S ribosomal protein S1	543.5
mtrC	542	C	T	T181I	Membrane fusion protein	525.5
HP0498	744	T	G	F248L	Sodium^−^ and chloride^−^ dependent transporter	597.5
proWX	1650	A	C	R550S	Osmoprotectant ABC transporter permease	642
HP0853	943	C	A	R315S	ABC transporter ATP-binding protein	441.5
hypD	474	G	A (stop)	W158X	Hydrogenase maturation factor	512
hefD	13	G	T	G5C	Outer membrane protein	583.5
hefD	82	A	G	M28V	Outer membrane protein	346
tolB	210	T	G	D70E	Translocation protein	705.5
rpsP	226	G	A	A76T	30S ribosomal protein S16	650
HP1181	826	A	G	I276V	Multidrug transporter	506
rplA	464	G	A	S155N	50S ribosomal protein L16	561.5
HP1220	661	G	A	A221T	ABC transporter ATP-binding protein	628
HP1220	662	C	T	A221V	ABC transporter ATP-binding protein	624.5
moaA	76	C	T (stop)	Q26X	Molybdenum cofactor biosynthesis protein A	528.5

^a^Nucleotide position was determined by the respective reference sequence from NC_000915

Variations were mainly concentrated in membrane proteins and ABC transport ATP-binding proteins. Membrane proteins of the RND family, including *mtrC* and *hefD*, were identified variations in *H. pylori* CLR-susceptible strains with mutation of A2143G. The variations of major facilitator superfamily of HP1181 and ABC superfamilies were found in both strains. Simultaneously, two terminate mutations were observed in Hydrogenase maturation factor (hypD) and molybdenum cofactor biosynthesis protein A (moaA).

## Discussion

CLR has been widely used as a first-line drug for *H. pylori* eradication therapy, and has achieved remarkable achievements for the past few decades [4, 30]. Although the mechanism of CLR resistance has been well illustrated, it is difficult to explain the bacterial antibiotic resistance of some strains with different genotype and phenotype. However, researchers were mainly committed to exploring the genes associated with CLR resistance [11, 17]. To our best knowledge, there is little attention on CLR susceptible gene. In this study, to characterize the variations of multidrug efflux transporter genes, we first complete the whole-genome sequencing of CLR-susceptible *H. pylori* with mutation of A2143G in the 23S rRNA.

In the present study, we applied HiSeq 2500 platform to generate sufficient reads for analyzing the whole genome sequences of *H. pylori*. The clean short overlapping reads after quality control were directly mapped against reference genome without assembly [31]. Although the coverage was not very high, the multidrug efflux transporter genes and 23S rRNA gene were well covered with depth of at least 300-fold. Because of genomic gaps, we didn’t choice to analysis of insertions or deletions (InDels).

To elucidate the differences in genotype and phenotype, the analysis of 23S rRNA gene mutation was carried out, and the point mutations at position 2143 were consistent with NGS. Consequently, NGS was a precise method to distinguish the point mutation in genome [22–24]. Overall, mutations of 23S rRNA were disorganized and unregulated. There were no significant differences between CLR phenotype-resistant strains and CLR phenotype-susceptible strains, and nor between CLR genotype-resistant strains and CLR genotype-susceptible strains. On the contrary, the mutation of T2182C was detected in all strains except for S7. Although gene mutation of T2182 was identified with low resistance level in previous study [32], this mutation was detected in most of the strains in China and the result of this discrepancy may be contribute to geographical and genetic factors [2].

In present study, to analyze the discrepancy between CLR-resistant strains and CLR-susceptible strains, the mutations of twenty-two multidrug efflux transporter genes were extracted. HP0605 knockout mutant presents more susceptibility to novobiocin and sodium deoxycholate [12]. Agreement with previous study, we found that the total SNVs of multidrug efflux transporter gene and the SNV of HP0605 were significant differences (P ≤ 0.05) between phenotype resistant and susceptible strains, while not been found in other genes [33]. Thus, we speculate the SNVs of HP0605 probably have an effect on *H. pylori* resistance to CLR. Unexpectedly, the SNVs of the RND family were significantly higher in mutant strain (A2143G) than in wild type. The different results mainly contributed to the two strains with different genotype and phenotype. We speculated that the 23S rRNA mutant strains with susceptible to CLR were initially resistant to CLR. Nonetheless, with the living environment and genetic changes, the phenotype of CLR has transformed. This can illustrate that these two strains exhibit more tendency to other strains with consistent genotype and phenotype in genetics.

Efflux pump systems have been identified in bacteria to be associated with antibiotic resistance [14–16]. The RND family, commonly used as a Gram-negative bacteria antibiotic study, is also used for *H. pylori*. Amsterdam et al. [12] revealed that more susceptible to metronidazole (MTZ) for HP0605 and HP0971 double-knockout mutant *H. pylori* strain. The expression of membrane proteins of the RND family has been a hot spot in the study of bacterial resistance. It has been proven that TolC and its homologues play an important role in molecules efflux, virulence and drug resistance [34]. *hefD* (HP0971) and *mtrC* (HP0606) are an outer membrane protein (TolC) and a membrane fusion protein (AcrA) of the RND family, respectively. The result of NGS has shown that there have three special mutations present in two CLR-susceptible strains with genotype of A2143G, and not in others. And also, this phenomenon has been found in outer membrane protein *hopG* (HP0254). Therefore, we attempt to speculate that the variations of outer membrane protein are associated with bacterial antibiotic resistance in *H. pylori*. Although the exact molecular mechanism of *H. pylori* with genotype of A2143G susceptible to CLR is unclear, our findings have indicated that the variations of membrane proteins possibly contribute to influence *H. pylori* resistance to CLR.

Of course, our research also has some limitations due to the problem of coverage and we did not analyze the impact of InDels on the experimental results. We empirically selected some genes that were associated with drug resistance for SNVs analysis. Indeed, we have characterized some special variations of HP0606 and HP0971 in CLR-susceptible strains with mutation of A2143G, and we believed that there have some other genes that regulate the susceptibility of bacteria to antibiotics. Undeniably, whole-genome sequencing of *H. pylori* provides a new way to solve the problem of bacteria antibiotic resistance.

## Conclusion

In this study, we successfully isolated two CLR-susceptible *H. pylori* strains with mutation A2143G of 23S rRNA gene. Genome variations were analyzed by whole-genome sequencing between twelve *H. pylori* strains with different CLR resistances. The data of sequencing suggested that the next-generation sequencing of clinical isolated *H. pylori* is a useful method for identifying genome variations. Analysis of multidrug efflux transporter gene mutation results indicated that membrane proteins of RND family possibly play an indispensable role in resistance to CLR. Further studies of *H. pylori* genomic variation should be paid more attention to disentomb potential gene associated with antibiotic resistance or susceptibility.

## Abbreviations

SNVs: single nucleotide variants
CLR: clarithromycin
RND: resistance-nodulation-cell division
NGS: next-generation sequencing
MFS: major facilitator superfamily
InDels: insertions or deletions
